# A Novel Secretory Poly-Cysteine and Histidine-Tailed Metalloprotein (*Ts*-PCHTP) from *Trichinella spiralis* (Nematoda)

**DOI:** 10.1371/journal.pone.0013343

**Published:** 2010-10-13

**Authors:** Georgi Radoslavov, Rositsa Jordanova, Denitsa Teofanova, Katya Georgieva, Petar Hristov, Marco Salomone-Stagni, Eva Liebau, Ilia Bankov

**Affiliations:** 1 Biochemistry Department, Institute of Experimental Pathology and Parasitology, Bulgarian Academy of Sciences, Sofia, Bulgaria; 2 European Molecular Biology Laboratory, Hamburg Outstation, Hamburg, Germany; 3 Institute of Zoophysiology, Westfälische Wilhelms-Universität (WWU), Muenster, Germany; Indiana University, United States of America

## Abstract

**Background:**

*Trichinella spiralis* is an unusual parasitic intracellular nematode causing dedifferentiation of the host myofiber. *Trichinella* proteomic analyses have identified proteins that act at the interface between the parasite and the host and are probably important for the infection and pathogenesis. Many parasitic proteins, including a number of metalloproteins are unique for the nematodes and trichinellids and therefore present good targets for future therapeutic developments. Furthermore, detailed information on such proteins and their function in the nematode organism would provide better understanding of the parasite - host interactions.

**Methodology/Principal Findings:**

In this study we report the identification, biochemical characterization and localization of a novel poly-cysteine and histidine-tailed metalloprotein (Ts-PCHTP). The native Ts-PCHTP was purified from T. spiralis muscle larvae that were isolated from infected rats as a model system. The sequence analysis showed no homology with other proteins. Two unique poly-cysteine domains were found in the amino acid sequence of Ts-PCHTP. This protein is also the first reported natural histidine tailed protein. It was suggested that Ts-PCHTP has metal binding properties. Total Reflection X-ray Fluorescence (TXRF) assay revealed that it binds significant concentrations of iron, nickel and zinc at protein:metal ratio of about 1∶2. Immunohistochemical analysis showed that the Ts-PCHTP is localized in the cuticle and in all tissues of the larvae, but that it is not excreted outside the parasite.

**Conclusions/Significance:**

Our data suggest that *Ts*-PCHTP is the first described member of a novel nematode poly-cysteine protein family and its function could be metal storage and/or transport. Since this protein family is unique for parasites from Superfamily Trichinelloidea its potential applications in diagnostics and treatment could be exploited in future.

## Introduction

Trichinosis, also called trichinellosis, or trichiniasis, is a parasitic disease caused by nematodes of the genus *Trichinella*. It is present worldwide and hundreds cases of human trichinellosis occur yearly. *Trichinella* is often referred to as the largest intracellular parasite. During its life cycle the parasite occupies two primary tissue sites, the intestinal epithelium (adults) and the skeletal muscle fibers (muscle stage larvae, L1). Trichinellids are unique since in both of these environments the nematodes occupy intracellular niches. Invasion of vertebrate striated muscle cells by larvae of *Trichinella spiralis* is accompanied by dedifferentiation of the occupied portion of host myofiber into a novel structure called the nurse cell-parasite complex. The nurse cells protect and nurtures the accommodated parasite during its long stay into the host muscle [1], [2]. In these parasitic nematodes specific metabolic pathways are modified to meet their actual physicochemical environment, the host cells. Different aspects of parasite's metabolism could be possible targets for chemotherapy and are object of intensive research in parasitology. Recent studies on *Trichinella* and closely related to them species, the parasitic worms from genus *Trichuris* (Trichuridae), are focused on differences between parasite and host biochemistry [1]. *Trichurids* are intracellular parasites only in the intestinal stage (adults) and untypical to *Trichinellids* have a free living development of the larval stages. Both families should be considered as occupying unusual niche for parasites within the phylum Nematoda [3], [4].

Species of genus *Trichinella* induce a wide range of changes in the host, but their nature and mechanisms are still poorly understood. Recently, a detailed proteomic analysis has been employed and identified number of proteins that act at the interface between the parasite and the host [5]. These proteins are able to modify the environment by modulation either of the host immune response or the host cell protein expression thus ensuring the survival of the parasite [6]. Circumstantial evidence implicates larval excretory-secretory (E-S) proteins to be involved in these processes and various of these proteins have been identified as antigens or playing a role in the nurse cell formation [5], [7]. It remains uncertain whether the E-S proteins from muscle larvae are active during the intracellular infection or expressed in preparation for the subsequent intestinal phase of infection. It is likely that the E-S proteins affect host cells and tissues metabolism but non-E-S proteins are likely engaged in internal reactions within the parasites [5]. There is still not enough information on the role and function of both of these protein groups, and especially on the non-E-S proteins.

E-S products from the *Trichinella* spp. are proteins comprises heat or cold shock proteins, endonucleases, proteinases, protein kinases, proteinase inhibitors, superoxide dismutases (SOD), glycosidases as well as many proteins with unrevealed function like the 43-, 53,- and 45 kDa glycoproteins [5]. Research on proteins mentioned above as well as other enzymes from *T. spiralis* was carried out to shed light on specific metabolic pathways of the parasite in order to identify potential drug targets for novel chemotherapies. As a result several enzymatic antioxidants or proteins involved in signal transduction pathways like protein prenyltransferases were highlighted and studied further for selective inhibition [8].

Many enzymes and other proteins from *T. spiralis* E-S and non-E-S products have been identified as metalloproteins that bind different divalent cations. These include serine proteinases and metalloproteinases, serine proteinase inhibitor (serpin) and copper-zinc SOD and manganese SOD [9], [10]. Numbers of metalloproteins identified in *Trichinella* are cysteine-rich and nematode specific. Some of them are proteins with zinc-finger motifs such as the cysteine-rich FYVE finger domain protein that coordinates two zinc atoms [11]. Others like *Ts*-CCG-1 and *Ts*-CCG-2 possess conserved nematode specific cysteine (Cys)-glycine (Gly) domains [12]. Another cysteine-rich protein found in the *T. spiralis* is a multi-domain cystatin-like protein. The latter has not assigned function yet and is part of the cystatin-like protein group that has no cysteine protease inhibitory activity [13]. However, the role of nematode proteins in metabolic pathways, transport and detoxification of metal ions is poorly investigated and more research in this direction is needed, especially since some metalloproteins could present novel drug targets.

We have identified and analyzed a novel 48 kDa poly-cysteine and histidine-tailed metalloprotein from *Trichinella spiralis* (*Ts*-PCHTP), specific for *Trichinella* parasites. We have defined its nucleotide and amino acid sequences, as well as its secondary structure, posttranslational modifications and tissue localization.

## Materials and Methods

### Parasites

*Trichinella spiralis* (T1 ISS03) was maintained by serial passage infections in Wistar rats [14]. The infective-stage muscle larvae were recovered from experimentally infected rats at 30 days post infections by a standard enzymatic digestion method and multiple precipitations according to a standard protocol [14] and stored at −70°C.

### Native protein purification

Native *Ts*-PCHTP was purified from muscle *T. spiralis* larvae as described for Ag-lbp55 and Ag-NPA-1 [15], [16]. The supernatant after 70% ammonium sulfate precipitation in 20 mM Tris buffer, pH 7.4 was further purified by anion exchange chromatography on DEAE-cellulose. Elution was performed with 10 mM Tris buffer, pH 6.5 at a gradient saline concentration 0–0.5 M fo NaCl. The bound and unbound DEAE-cellulose fractions were subjected to size exclusion chromatography on Superdex 75 or HPLC BioSuite^tm^ 125, 10 µm SEC column (Waters). The elution was performed with 10 mM Tris buffer, pH 7.4.

*Ts*-PCHTP determined sequence revealed that the protein contains a “natural” poly-histidine (poly-His) tail. Thereby *Ts*-PCHTP was successively purified also from muscle larvae crude extract by Ni-affinity chromatography with a His Trap kit (GE Healtcare BioSciences) following the manufacturer's protocol.

To avoid degradation of the native protein, all steps were performed at 4°C and in the presence of a protease inhibitor mixture (Complete Protease Inhibitor Mixture; Roche).

The molecular size and purity of the protein was determined by SDS-PAGE. The protein was identified and further analyzed by tryptic in gel digestion (Trypsin Gold protocol, Promega) and MALDI-TOF mass spectrometry. MALDI-TOF analysis of the obtained peptides was performed on Voyager MALDI-TOF MS (Applied Biosystems) and the data were analyzed with PEPTIDE MASS software MASCOT, FindPept tool, PeptideMass and GlycoMod (www.expasy.org/) [17], [18], [19].

The protein concentration was determined initially by the method of Bradford [20]. After obtaining *Ts*-PCHTP amino acid sequence, it was also determined spectrophotometrically using a molar extinction coefficient of 1,332 M^−1^ at 280 nm as calculated on the basis of the aromatic amino acid of 10 Tyrosine (Tyr) and 8 Tryptophan (Trp) residues with ProtParam tool [21] (www.expasy.org/).

### Circular dichroism (CD)

CD spectra were recorded in 10 mM Tris buffer, pH 7.5, 20°C, using a Jasco Model 715 automatic recording CD spectrophotometer with a thermostatically controlled cell holder. A fused quartz cell with a path-length of 0.1 cm was used. The spectra, measured in the far UV-region 190 nm–260 nm, were averaged of four scans and were corrected by subtracting the baseline of the buffer. They are reported as Delta Epsilons (Δε) in degrees mdeg M^−1^cm^−1^. Spectra subtraction, normalization and smoothing were performed using JASCO CD J-715 data manipulation software and the analysis of the data was carried out with the programs SELCON and CONTIN [22], [23].

### Nucleotide and Amino Acid sequences

The N-terminal sequence of the purified *Ts*-PCHTP was determined by automated Edman degradation after protein bands were cut out of the SDS-PAGE gel. Based on the N-terminal sequence obtained (LPGLGCGWTVLQEVVK) two EST (Expressed Sequence Tags) fragments from *T. spiralis* (pc20c06.y4 and pc32b04.y1) were identified in the EST database (www.nematode.net) [24]. Specific oligonucleotides were designed to amplify the missing 5′ and 3′ ends of the cDNA from the *ts*-pchp gene (Table 1, Table S1). Total RNA from muscle larvae was obtained by homogenization using Trizol Reagent (Invitrogen) according to the manufacturer's instructions. Reverse transcription was performed with 4 µg of RNA, oligo (dT) primer and M-MuLV Reverse Transcriptase (Fermentas).

**Table 1:** Specific oligonucleotide primers used to obtain full length cDNA sequence.

Forward Primers	
S1	ATGGCTTTCTCAACTATTGT
S11*	GGGA***ATTCCATATG***GCTTTCTCAACTATTG
S2	GTCGGCCGACACATGTCCCG
S22*	GGA***ATTCCATATG***AACAAAATTTCGTCGGCCGA
S3	CTGCGGTTGGACAGTTTTGC
S5	AGACCTGACATACCTGAATA

Reverse Primers	
AS1	GGGACATGTGTCGGCCGACG
AS2	GAAACTGCAATGACCACAAG
AS3*	CGCGG***ATCCTT***ATCAATGATGATGATGATGATGATG

With asterisk are shown primers used for expression vector pJC20; sequences in bold and italic are BamHI and NdeI restriction sites.

In order to obtain the missing 5′ and 3′ ends of the gene cDNA fragments were cloned, isolated and sequenced by the 5′- and 3′-RLM-RACE method using FirstChoice® RLM-RACE Kit (Ambion, Applied Biosystems) according to the manufacturer's manual. Oligonucleotide primers - S1, S2, AS1 and AS2 (Table 1) were designed based on the *T. spiralis* ESTs, pc20c06.y4 and pc32b04.y1 (www.nematode.net). PCR products were cloned in pCR2.1-TOPO vector using TOPO® TA Cloning® Kit (Invitrogen) and transformed into *Escherichia coli* DH5α cells according to the manufacturer's manual.

### Production of recombinant *Ts*-PCHTP

Several primers were designed for *E. coli* expression of the *Ts*-PCHTP coding region, S11 - encoding the first six amino acids including the ATG start codon, S22 - encoding the first six amino acids after signal peptide including the ATG start codon and the AS11 - encoding the last seven amino acids including the TGA - termination codon (Table 1). They were used to perform a PCR with reverse transcribed RNA of *T. spiralis* as a template. The sense primers S11 and S22, contained an NdeI restriction site and the antisense primer AS3 a BamHI restriction site to ensure directed cloning into the expression vector pJC20 [25]. The construct was then transformed into *E. coli* BLD (DE3) cells and used for the expression of the recombinant protein. The expression induction was utilized with final concentration of 1 mM IPTG for 4 hours at 37°C. Cells were lysed through sonification on ice. The soluble fraction was used for purification of recombinant protein. Recombinant *Ts*-PCHTP was purified as described for the native protein by Ni-affinity chromatography with a His Trap kit (GE Healtcare BioSciences).

### Deglycosylation

To investigate the existence of asparagine-linked glycan chains on *Ts*-PCHTP purified native protein was treated with N-Glycosidase F (N-Glycosidase F Deglycosylation Kit) according to the manufacturer's manual (Roche). Following deglycosylation, treated and non-treated protein extracts were loaded on SDS-PAGE and visualized with Coomassie blue staining.

### Metal binding properties

A PicoTAX-Automatic apparatus has been used for Total Reflection X-ray Fluorescence (TXRF) experiments (Röntec, Berlin, Germany). The PicoTAX is a cooling-free portable bench-top device. It operates with an air-cooled Mo based tube working at 40 KeV, 1 mA. The X-ray beam is focused by a Ni/C-multi layer monochromator. The detector is a Peltier-cooled XFlash, 10 mm^2^ with a resolution of 160 eV. Access to the instrumentation was kindly provided by Peter Freimann of the “Bundesamt fuer Seeschiffahrt und Hydrographie”, Hamburg). Scandium and gallium have been used as internal standards (ICP-Standard Gallium 1.000 g/L, ICP-Standard Scandium 1.000 g/L Bernd Kraft GmbH). Using these standards, TXRF gives micro-molar range element quantification for first row transition metals. The samples were mixed with a solution containing the standards so that the final concentrations of scandium and gallium were respectively of 20 mg/ml and 2 mg/ml. The specimen for analysis were prepared depositing 5 µl of solution at the centre of round-shaped ultraflat plexiglass sample trays (1.5 mm Ø×3 mm, manufactured by the EMBL-Heidelberg work shop, Heidelberg, Germany) and let them dry at 37°C. Every specimen has been measured for 5000 seconds. Each sample has been analyzed in triplicate and metal quantification of a given sample has been always compared with the metal quantification of the pure buffer. The data were analyzed with the program PyMCA [26].

### Antibodies selection and Western blotting

Five hundred µg of purified native *Ts*-PCHTP were used to raise antibodies against the protein in rabbit (Institute of Experimental Pathology and Parasitology, Bulgaria). The final bleed was used to analyze the native and recombinant protein at a dilution of 1∶10 000. After discovering that the native protein contains poly-histidine tail the monoclonal Anti-polyHistidine−Alkaline Phosphatase antibody (Sigma-Aldrich) was used for identification as well.

For immunoblotting *T. spiralis* soluble proteins were separated by SDS/PAGE on 12% gel and transferred to nitrocellulose membranes [27]. Membranes were blocked with 2.5% bovine serum albumin (BSA) (Sigma-Aldrich) in phosphate buffer saline (PBS) and subsequently incubated with the primary antibody at a dilution of 1∶10 000. After extensive washing with PBS/Tween, the membranes were incubated with an alkaline phosphatase-conjugated ProteinA-secondary antibody (Sigma-Aldrich) and developed with nitro blue tetrazolium and 5-bromo-4-chloro-3-indolyl phosphate.

### Immunohistology and immunogold Transmission electron microscopy (TEM)

Muscle samples with encapsulated *T. spiralis* muscle larvae (capsule stage and after pepsin digestion) were fixed in 4% paraformaldehyde in Tris-buffered saline (TBS) pH 7.2. Following fixation, samples were rinsed 3 times for 10 min in TBS, dehydrated on ice in a graded series of ethanol and embedded in LR white H acrylic resin (Sigma-Aldrich), according to the manufacturer's recommendations. Antisera against *Ts*-PCHTP were raised in rabbit using a standard immunization protocol as mentioned above. The preimmune serum was used as a control. For light microscopy either anti-*Ts*-PCHTP serum or monoclonal anti-poly-His antibody (Sigma-Aldrich) were used as a primary antibody at dilutions of 1∶500. Immunolocalization on the light and on the electron microscopical level was performed as described previously [16].

For light microscopic immunohistology an indirect immunofluorescence technique was used. Semi thin sections were mounted on glycerine-albumine coated glass cover slips. All samples were washed preliminary with TBS three times and three times between all incubations. The first incubation was performed with goat serum (1∶500 diluted in TBS) for 2 h at room temperature to prevent nonspecific binding reactions. Then the samples were incubated for 2 h with rabbit polyclonal antiserum raised against native *Ts*-PCHTP in 1∶500 dilutions. The next incubation was for 2 h with 1∶500 FITC- immunostaining according to the manufacturer's recommendations (Sigma-Aldrich). After washing the samples were examined on a Leica DM 5000B microscope using a filter specific for fluorescein. As controls the primary antibodies were replaced by the normal rabbit preimmune serum.

For immunogold TEM the ultrathin sections were collected on nickel grids (300 mesh), which were posed on drops of 5% BSA in TBS (Sigma-Aldrich) for 2 h. Then the sections were incubated with the same primary antibodies as in the immunofluorescence assay in 1∶500 dilutions for 2 h. After washing, the sections were treated with Protein A Gold 20 nm (Sigma-Aldrich), diluted 1∶10 in TBS for 2 h. The sections were washed with buffer and counterstained in 1% aqueous uranil acetate and lead citrate for 30 min and 1 min respectively, and examined by a JEOL 1200 EX transmission electron microscope. Controls included the omission of the primary antibodies from incubation steps.

### Sequence analysis and structure prediction

BLAST [28] and PSI-BLAST [29] were used for local sequence database searches and alignments were calculated using ClustalW2 [30].

#### Protein sequence analysis and structure prediction

Sequence analysis and secondary structure predictions for the protein were performed with programs available on the ExPaSy molecular biology server (us.expasy.org/tools/); the molecular mass and isoelectric point (pI) of the protein were estimated with the ProtParam program; Gor4 [31] Jpred [32] and 3D-PSSM programs [33] were used for secondary structure predictions. Searches of predefined sequence families were performed using the web interfaces to Pfam [34], the conserved domain database [35] and SAM-T02 [36], [37].

#### DNA sequence analysis and structure prediction

The ssDNA secondary structure folding prediction was performed with the mfold v.3.2 software (http://www.bioinfo.rpi.edu/applications/mfold) [38], [39]. Folding predictions were prepared for the sense and antisense strand with varying folding temperatures and ionic conditions.

## Results

### Protein purification

**Figure 1:**
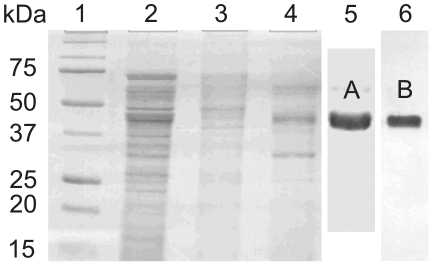
Purification of native *Ts*-PCHTP with anion exchange chromatography followed by size exclusion chromatography. (A) and Ni-NTA agarose purification (B). 1) markers; 2) soluble fraction after ultracentrifugation; 3) supernatant after 70% saturation with ammonium sulfate; 4) unbound fraction of anion exchange chromatography on DEAE-cellulose; 5) *Ts*-PCHTP after size exclusion chromatography on Superdex 75; 6) *Ts*-PCHTP after Ni-NTA chromatography. Proteins were visualized by Coomassie Blue staining.

Native *Ts*-PCHTP was purified from *T. spiralis* muscle larvae. Since the amino acid sequence was unknown the initial purification procedure included ammonium sulfate precipitation of the soluble protein fraction, anion exchange chromatography on DEAE-cellulose and size exclusion chromatography on Superdex 75 (Figure 1). The native protein was isolated as a single band corresponding to the molecular mass of the monomer of approximately 48 kDa. Peptide mass fingerprint method was used to identify the purified protein initially. The mass spectrum of the tryptically cleaved *Ts*-PCHTP was compared to known nematode and other proteins using the software MASCOT (http://www.expasy.org/) but no homology was observed.

After obtaining the amino acid sequence as described below, it became evident that *Ts*-PCHTP contains seven consecutive histidine residues at the C-terminal. This allowed alternative purification of the native protein by Ni-affinity chromatography following either ammonium sulfate precipitation (Figure 1) or directly from homogenate. The purified native *Ts-*PCHTP represented 0.1%–0.2% of the soluble protein fraction and was one of the most abundant proteins in *T. spiralis* muscle larvae. HPLC size exclusion chromatography and MALDI-TOF analysis demonstrated the homogeneity of the purified native *Ts*-PCHTP. The molecular weight as determined by mass spectroscopy is 48105 Da, which is in good agreement with the calculated mass of 47744 Da. Western blot analysis showed that different primary antibodies (antiHis- and polyclonal anti *Ts*-PCHTP rabbit antibodies) reacted against the purified protein fraction as well as the *Trichinella* protein homogenate and unspecific reactions were not observed.

*Ts*-PCHTP coding regions with and without signal peptide were cloned into an expression vector pJC20 and the constructs were transformed into *E. coli* strain BLD (DE3). Recombinant *Ts*-PCHTP was purified from soluble bacterial homogenate by Ni-affinity chromatography. Western blot analysis with antiHis- and polyclonal anti-*Ts*-PCHTP rabbit antibodies showed immunogenic traits of the recombinant protein fractions.

### Nucleotide sequence

**Figure 2:**
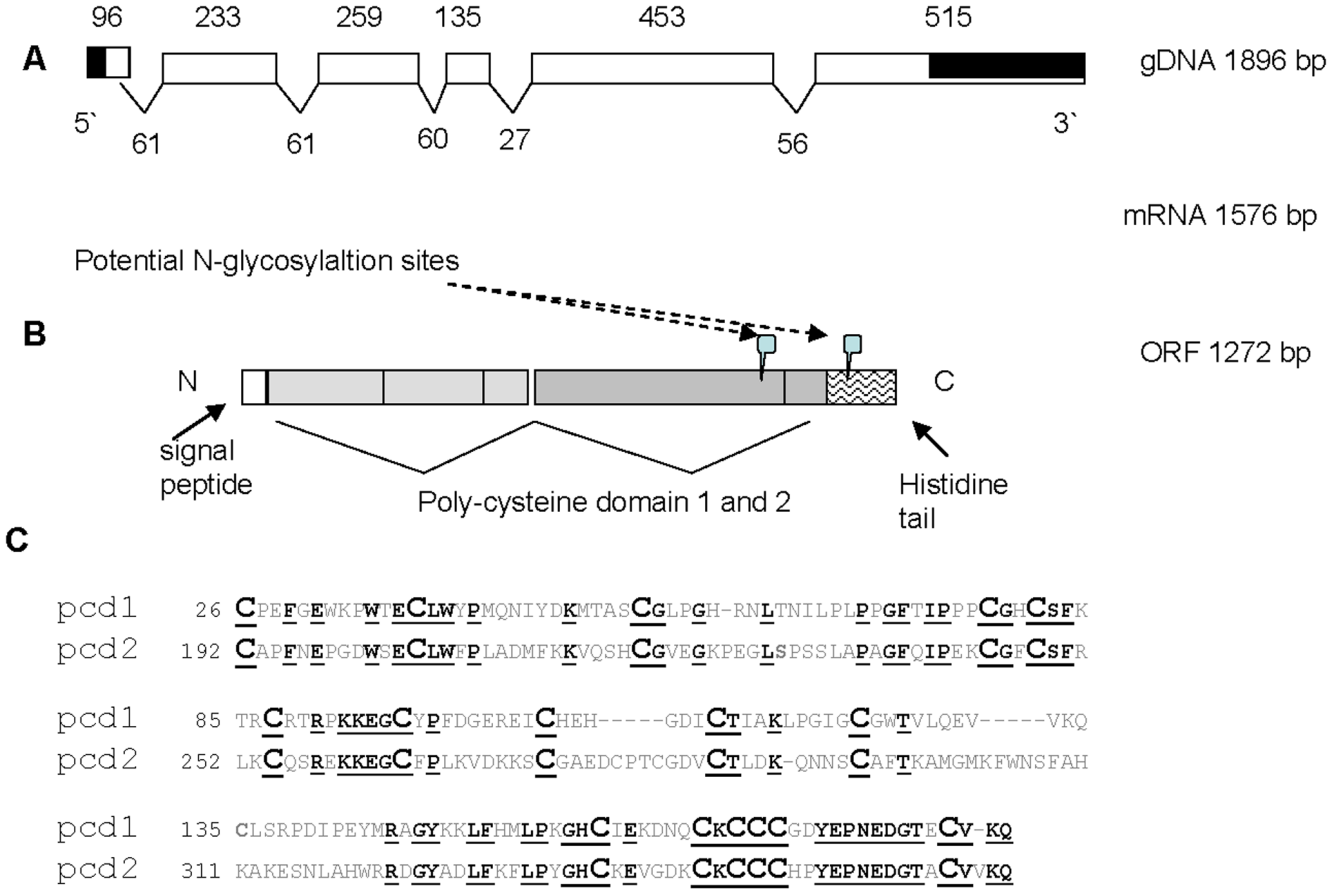
Organization of *ts*-pchtp gene and protein chain. A) Comparison between *Ts*-PCHTP cDNA and whole genome shotgun sequence, ABIR01001777 (start 65194–end 67089) from *Trichinella spiralis* showed 6 exons and 5 introns of the 1896 bp gene. B) Open reading frame of translated protein sequence contains signal peptide (exon 1), two homology poly-cysteine domains (exon 2–4 and exon 5–6) and histidine tail on C-term end (exon 6). The 2 potential glycosylation sites are shown. C) Comparison between poly-cystein domain 1 and 2. Data showed 38.5% identity at 174 amino acid. Conservative cysteine residues are in bold.

The first amino acid sequence fragment LPGLGCGWTVLQEVVK was obtained by N-terminal protein sequencing of the native protein. Database mining found this fragment in two *T. spiralis* ESTs, pc20c06.y4 and pc32b04.y1 (www.nematode.net), [24]. The 5′- and 3′- ends of the *ts-*pchtp mRNA were determined by RLM-RACE method. Additionally, oligonucleotide primers S1, S2, AS1 and AS2 were designed (Table 1), based on the *T. spiralis* ESTs pc20c06.y4 and pc32b04.y1. Obtained PCR products were cloned into pCR2.1-TOPO vector and sequenced. The new fragments were compared with other *T. spiralis* ESTs (www.nematode.net), [24] as well as the whole genome shotgun sequence of the *T. spiralis*, GenBank™ accession number ABIR01001777 (Genome sequencing project at Washington University School of Medicine). As a result the full sequence of *ts-*pchtp gene was identified (Figure 2, Figure S1).

The nucleotide sequence on the mRNA consists of 1576 bp and contains both the 5′- and the 3′ noncoding region. The transcript has a single open reading frame from 1272 bp, which translates into 424 amino acids that compose the full-length protein (Figure 2). The *ts-*pchtp sequence has been deposited in the GenBank™ under accession number GQ497342. Comparison between *ts-*pchtp cDNA and the whole genome shotgun sequence ABIR01001777 showed that the gene consist of six exons and five introns of 1896 bp (65194 bp to 67089 bp of ABIR01001777). The full gene organization is shown on Figure 2A. The 5′ UTR includes a part from the first exon (position 1 bp to 42 bp) and the 3′UTR from the sixth exon (position 1637 bp to 1896 bp). Additionally the mature mRNA sequence does not contain a nematode specific splice leader [40].

Some unusual features of the cDNA and gDNA fragments coding *Ts*-PCHTP were revealed during the PCR and sequencing procedures. The PCR products of the full ORF loaded on 1% agarose gel run as three significantly smaller fragments with mass corresponding to app. 800 bp, 600 bp and 300 bp rather than the predicted 1272 bp (primers S11/AS11, Table 1) or 1217 bp (primers S22/AS11, Table 1). Further cloning and sequencing analysis of these fragments showed that the ∼800 bp product contains the full ORFs of 1272 bp and 1217 bp respectively. PCR products of 600 bp and 300 bp sizes appeared to be different self-spliced fragments in which the middle part of the gene was missing (112 bp to 1121 bp, 149 bp to 1121 bp and 245 bp to 1002 bp) (Figure S2). On the other hand, the products amplified with the internal primer S3 or S5 and the antisense primer AS3 (Table 1) gave single PCR fragments of the correct molecular weight. Additionally, some PCR fragments also showed unusual splice variants like direct or inverted repeats in the 3′ UTR and recombination in the pCR2.1-TOPO plasmids, used for the cloning (data not shown).

A possible explanation for these observations is that the full length ORF can form stem-loops (hairpin loops) in a single-stranded DNA conformation as a result of several inverted repeats of the *ts-*pchtp gene (Figure S2). The ssDNA secondary structure folding was predicted with the mfold v.3.2 software [38], [39]. The results show that ssDNA of the *ts-*pchtp forms a bifurcated structure and over 20 single and external stem-loops similar to structures of the DNA/RNAzymes [41]. PCR amplification with the internal primers S3 and S5 disrupt important features of the splicing regions and thus these fragments do not have a secondary structure. Potential self-cutting rybozyme properties of the *ts-*pchtp mRNA were also identified in one fragment cloned into the expression vector pJC20 consisting of the full ORF of the *ts-*pchtp containing intron-1. *E. coli* BLD (DE3) cells transformed with this plasmid produced a recombinant protein which was identical to the molecular mass of the native *Ts*-PCHTP and also displayed the same antigenic properties (data not shown).

### Amino acid sequence

The amino acid sequence of *Ts*-PCHTP starts with a predicted signal peptide of 18 amino acid residues that is located on exon 1 and indicates an extracellular localization (Figure 2B, Figure S1).

Analysis of the primary structure suggests that the protein contains two homologous **p**oly-**c**ysteine **d**omains (pcd) pcd-1 and pcd-2 and a histidine tail with seven consecutive histidine residues at the C-terminus (Figure 2B,C). The protein sequence contains two potential N-glycosylation sites at positions 291 and 396, respectively. The calculated molecular mass of the native protein is 47744 kDa, a value similar to the experimentally obtained data. Peptide mass fingerprint analysis by software FindPept and FindMod (http://www.expasy.org/) of the peptides mass derived after tryptic digestion on the native protein corresponded to the acquired *Ts*-PCHTP sequence (Table S2).

The two pcd domains in the protein have 38.5% identity. The cysteine residues are some of the most abundant residues in *Ts*-PCHTP, 36 residues or 8.8% of the total amino acid content. The first pcd-1 domain (residues 26 to188) is composed from exons 2, 3 and 4, while the second pcd-2 (residues 192 to 363) from exon 5 and partially exon 6. The cysteine residues are in conserved positions between the two pcd domains (Figure 2C). A consensus motif (***C***x_11_ ***C***x_15_ ***C***Gx_15–16_Gx_6_ ***C***Gx***C***x_5_ ***C***x_7_G***C***x_9_ ***C***x_6–11_ ***C***x_8–9_ ***C***x_24–29_Gx_10_GH***C***x_6_ ***C***K***CCC*** **[**G/H**]**x_7_Gx_3_ ***C***) was identified from amino acid alignment among the pcd domains derived from available nematode sequences (Figure S3).

*Ts*-PCHTP also contains 26 histidine residues or 6.1% of the amino acid content. 14 of these are located in the C-terminal domain and 7 form an entire block at the end of the sequence, i.e. a poly-histidine tail.

Up to date only a few putative proteins with a histidine tail were reported such as the membrane Zinc transporter zitB from *E. coli* (P75757, Q8X400) and *Shigella flexneri* (Q83SA2), the cadmium, cobalt and zinc/H(+)–K(+) antiporter from *Bacillus* sp. (O07084, A7Z1S6), the 60 kDa chaperonins from *Mycobacterium* sp. (A4TEN6, P60545, A0QKR2) and from *Corynebacterium* sp. (Q8NSS0, A4QBU0, Q6NJ37), the ferrochelatases from *Lyngbya* sp. (A0YWB0) and *Nodularia spumigena* (A0ZDX2), the cobalamin (Vitamin B12) biosynthesis CbiX protein from *Halorhodospira halophila* (A1WWQ5), a cation transport protein (A1IW34) from *Yersinia enterocolitica* [42] and etc. However, most of these proteins are hypothetical or predicted. Unsurprisingly the hystidine rich part in the C-term of the protein shows partial homology with all hystidine rich proteins (hrg) as well.

**Figure 3:**
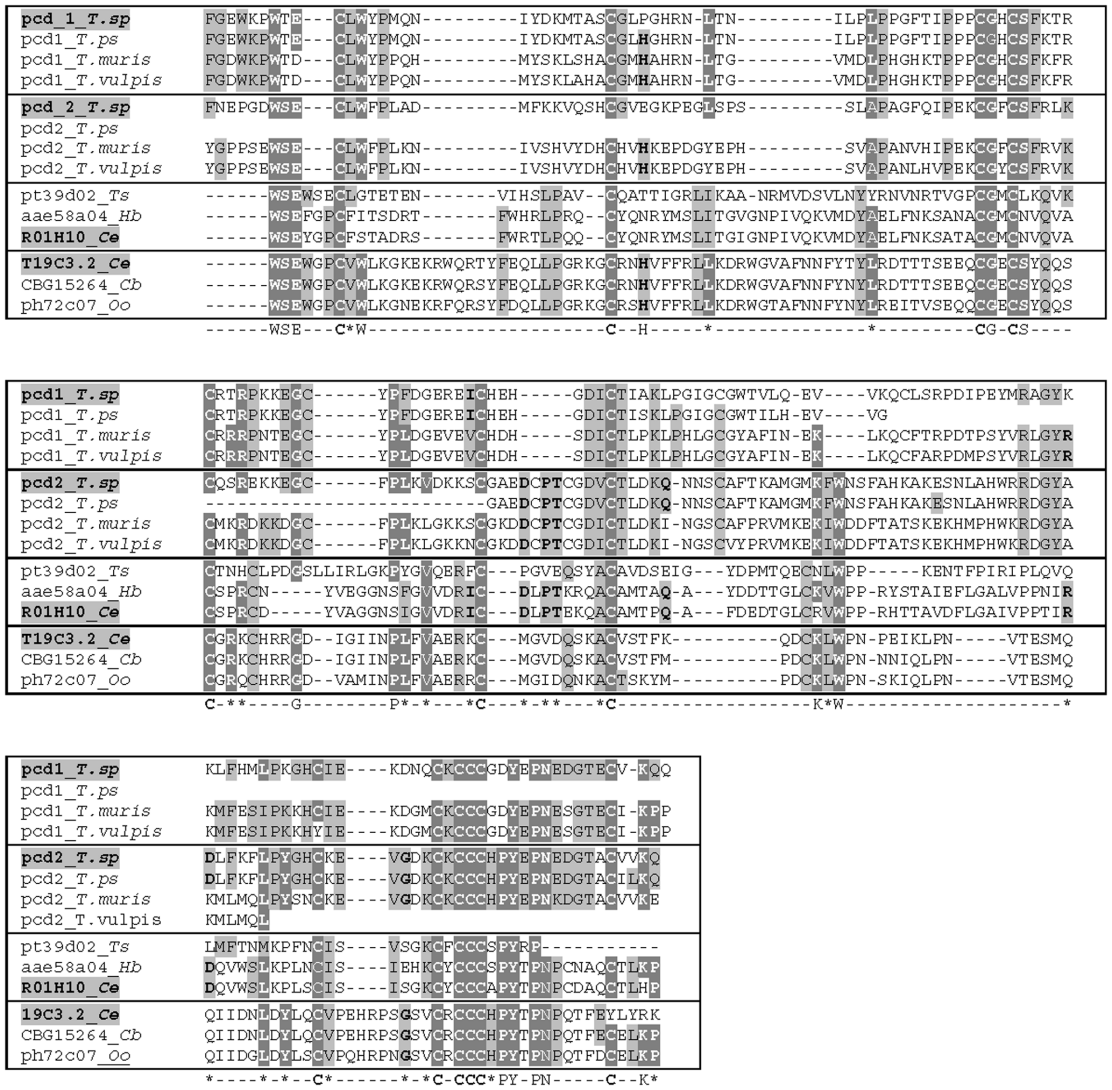
Comparison of the *Ts*-PCHTP pcd domains with *Caenorhabditis elegans* proteins and other nematode ESTs sequences. Bordered groups **pcd-1_T.sp** and **pcd-2_T.sp** contains pcd domains of the *Ts*-PCHTP and related ESTs (Table S1); bordered group **R01H10.4** contains ESTs pt39d02.y1 (*Trichinella spiralis*) and HTAB-aae58a04.b1 (*Heterorhabditis bacteriophora*); and bordered group T19C3.2 (YSV2_CAEEL) contains EST ph72c07.y1 (*Ostertagia ostertagi*) and hypothetical protein CBG15264 (*C. briggsae*). Homologous amino acids residues among groups are in grey boxes. Amino acids residues homologous for all proteins are shown with white letters.

A simple search in the non-redundant sequence database using BLAST or PSI-BLAST [28], [29] finds no homologues of *Ts-*PCHTP. There is only a partial similarity (∼25%) with two groups of close to *Caenorhabditis* nematode specific poly-cysteine proteins - 31 kDa protein T19C3.2 (YSV2_CAEEL) and 27 kDa protein R01H10.4 (Figure 3). The group related to the latter includes EST sequences from *T. spiralis* pt39d02.y1 and *Heterorhabditis bacteriophora* HTAB-aae58a04.b1. The former group includes EST sequences related to 31 kDa protein T19C3.2 like ph72c07.y1 and ph83d01.y1 from *Ostertagia ostertagi* and hypothetical protein CBG15264 from *C. briggsae*. The homology between these protein groups and *Ts-*PCHTP was mainly in the region of the motif ***C***x***CCC***[HPY]x[PN]x[PN]x5**C** and some other neighboring cysteine residues throughout the poly-cysteine domains 1 and 2 (Figure 3). Searching one set of precomputed domain profiles [35] or hidden Markov models [36], [37] with *Ts*-PCHTP provided no hits of significance.

However, the search against the nematode EST database (www.nematode.net) showed homology with sequences from *Trichinella pseudospiralis*, *Trichuris muris* and *Trichuris vulpis* (Table S1). Since the genus *Trichinella* is a monophyletic lineage in the Trichinellidae, which diverged 275MY (Permian) from the putative sister Trichuridae, this is not surprising [43]. On the basis of *Ts-*PCHTP sequence we composed *in silico* full hypothetical protein sequences from these EST fragments. Alignment with these sequences showed strong homology in the poly-cysteine domains, histidine tail and the conserved positions of all the cysteine residues (Figure 3, Figure S3). This alignment suggests that *Ts-*PCHTP might be a member of a novel PCHTP protein family, specific for the Superfamily Trichinelloidea. Additionally the signal peptide sequence (exon 1) differs in *Trichinella* and *Trichuris* species.

### Glycosylation of *Ts*-PCHTP

**Figure 4:**
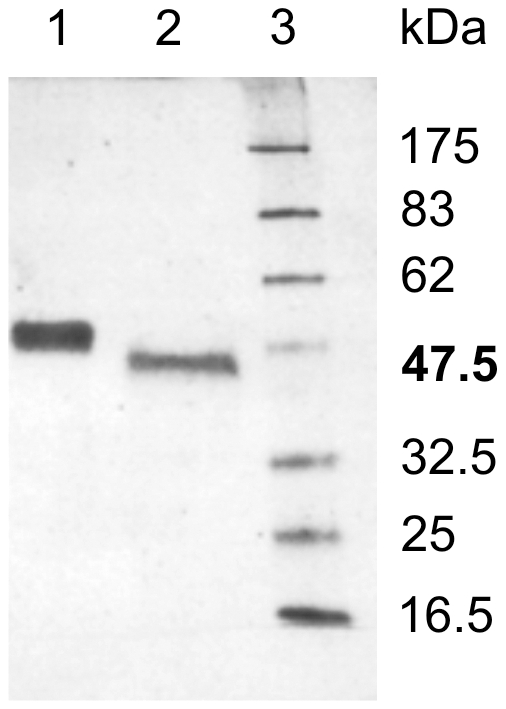
Deglycosylation native *Ts*-PCHTP with N-Glycosidase F. 1) eluted protein fraction after Ni-NTA chromatography; 2) deglycosylation with N-Glycosidase F; 5) marker. Proteins were visualized by Coomassie Blue staining.

There are some potential glycosylation sites in *Ts*-PCHTP sequence. The possibility of post-translational N-glycosylation of the native protein was investigated by treating it with the endoglycosidase *N*-glycosidase F. Figure 4 shows a SDS-PAGE in which the native *Ts*-PCHTP (Figure 4, line 1) and an enzymatically cleaved protein (Figure 4, line 2) are visualized. The presence of oligosaccharides in the protein molecule is clearly shown by the observed size differences. As already mentioned, the software NetNGly [44] predicted one *N-*glycosylation site at position 61 and two potential sites at positions 291 and 397 respectively. MALDI-TOF analyses of the peptides obtained after tryptic digestion using the software GlycoMod (http://www.expasy.org/) (Table S2) identified potential glycosylation modifications at residues 161 and 291 but not at residue 61. The type of the glycosylation chain was not investigated.

### Secondary structure

The sequence of *Ts*-PCHTP was given to several secondary structure prediction programs using their web interfaces [31], [32], [33]. Almost every region with any predicted secondary structure was most likely to be β-stranded. GOR4 method [31] predicted 23.82% β-strand, 65.80% random coil and 10.38% α-helix, while Jpred 3 [32] and 3D-PSSM [33] methods predicted 28.3% α-helix (Figure S1). The majority of α-helical structures are located predominantly at the end of the pcd domains and in the C-terminus of the protein. Studies on the secondary structure by circular dichroism (CD) confirmed that the overall structural content of *Ts*-PCHTP is predominantly β-stranded (Figure S4). The CD measurements in the far UV region (190 nm–260 nm) of *Ts*-PCHTP showed 44.4% beta structures, 21.1% alpha helices and 35.8% random coil as calculated by Selcon [22].

### Metal binding properties

**Figure 5:**
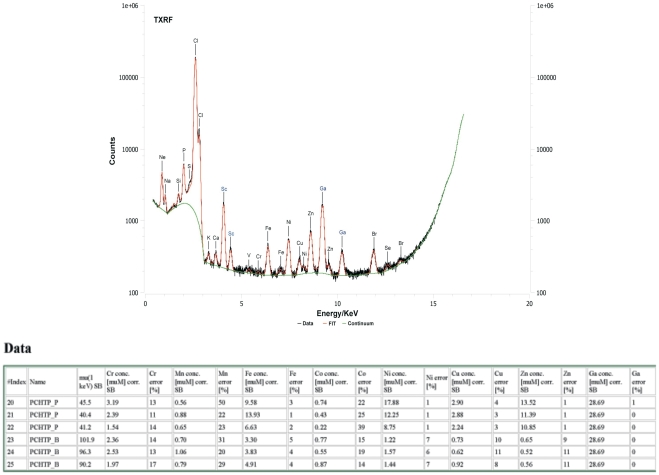
Total Reflection X-ray Fluorescence (TXRF) of the native *Ts*-PCHTP. 1 M of the native *Ts*-PCHTP bind about 1.5 M Fe; 2 M Zn and 2 M Ni.

The native *Ts*-PCHTP was investigated by Total Reflection X-ray Fluorescence (TXRF) for transition metal quantification. The analysis showed that the protein buffer alone had no metal contamination, which is indicative of a good preparation. Here, the transition metals are present in traces at what is considered a basal level. In comparison, the protein solution revealed that *Ts*-PCHTP was present together with a significant concentration of iron, nickel and zinc at an estimated protein:metal ratio of 1∶1.5, 1∶2.0 and 1∶1.9, respectively. Nickel might be an artifact of the purification procedure, rather than native ligand, but the Zn and Fe are likely to be native ligands of *Ts*-PCHTP. No significant amounts of Cu, Co, and Mn were detected in association with the protein (Figure 5). These results, corroborated by the presence of a natural poly-histidine sequence and of two pcd domains strongly support the hypothesis that *Ts*-PCHTP is a metalloprotein.

### Immunohistology and immunogold TEM

**Figure 6:**
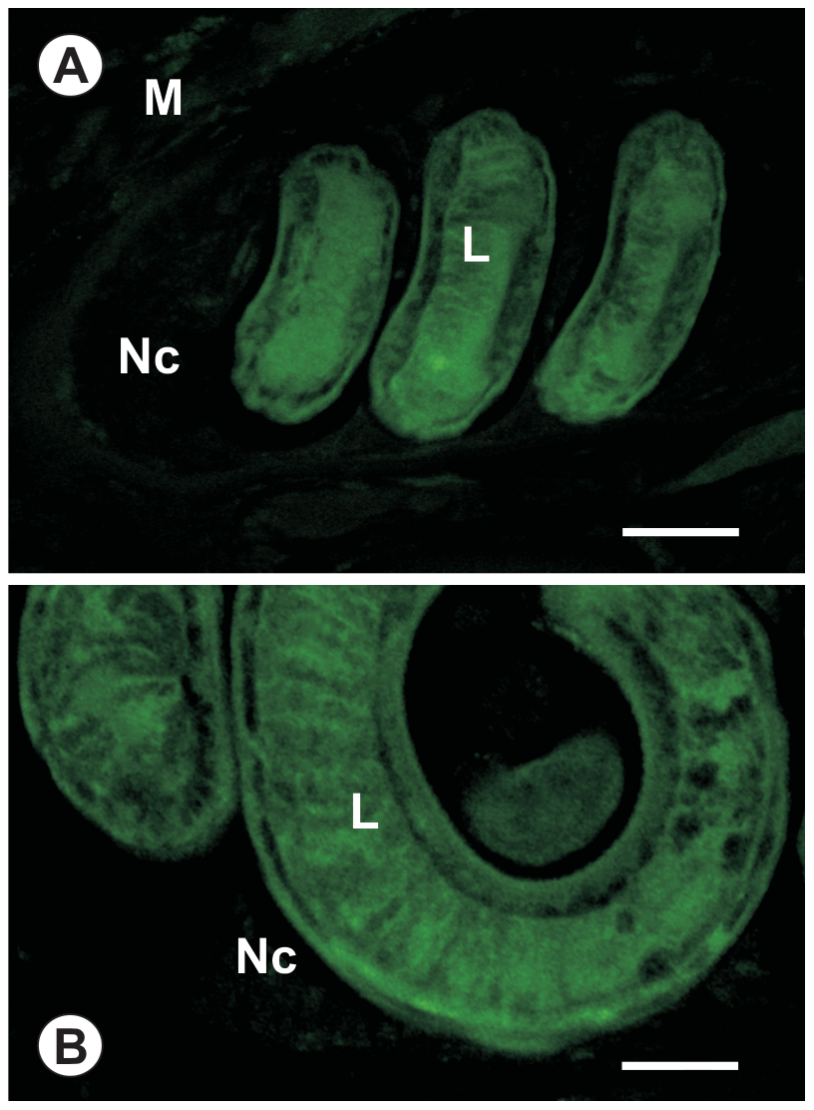
Immunofluorescent localization of *Ts*-PCHTP in *T. spiralis* larvae. (A) The intense staining of the encapsuled larva (L) after reaction with polyclonal serum. Nc - nurse cell, M - host muscle. (B) Labeling of the *Ts*-PCHTP in encapsuled larva (L) with anti-histidine antibody. Scale bar: A, B - 60 µm.

*Ts*-PCHTP was localized by indirect immunofluorescence technique in different developmental stages of *T. spiralis*, encapsulated muscle larvae and larvae after enteral stage after host infection. Two different antibodies against *Ts*-PCHTP were used - polyclonal anti-*Ts*-PCHTP and anti-histidine antibody. The reaction with the polyclonal serum gave stronger reaction compared to the anti-histidine antibodies (Figure 6). The results show significant staining of the cuticle as well as all tissues of the encapsuled larvae (Figure 6) but no fluorescence was detected into the nurse cells or the host striated muscle fibers, indicating that *Ts*-PCHTP is not excreted and thus no part of the E-S products. A similar staining pattern was observed after pepsin digestion of the larvae, a procedure that mimics the beginning of the enteral stage after host infection (Figure 6). Fluorescence was not observed in the control samples (data not shown).

**Figure 7:**
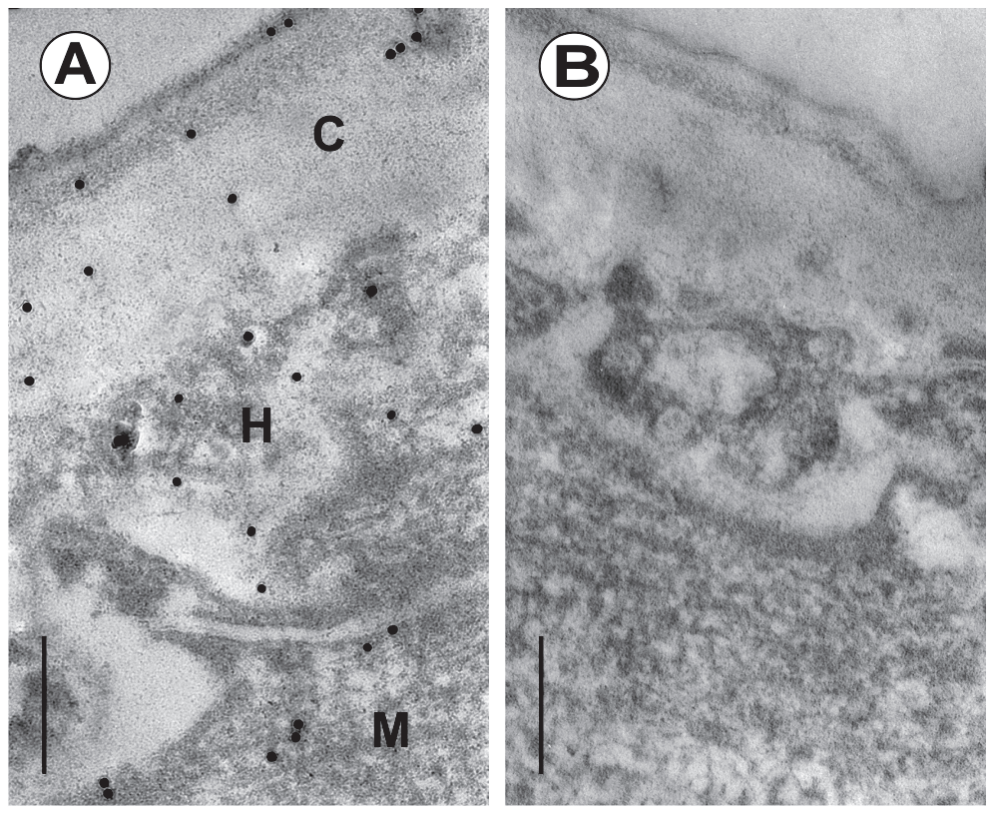
Immunogold electron microscopic localization of *Ts*-PCHTP in *T. spiralis*. (A) Strong accumulation of gold particles in the cuticle (C), hypodermis (H) and muscle (M) layers, revealed by polyclonal serum. (B) Control sample. Scale bar: A, B - 3 µm.

At ultrastructural level *Ts*-PCHTP was localized using the same antibodies and protein A-gold. Gold particles were detected in the cuticle, hypodermis and somatic tissues of the worm as shown on Figure 7A. The results support the immunofluorescence data and *Ts*-PCHTP was not detected in the nurse cells and host tissues. The binding of the primary antibodies was highly specific as demonstrated by the negative results of the controls (Figure 7B).

## Discussion

The current study describes the identification and characterization of a novel secretory 48 kDa metalloprotein *Ts*-PCHTP (GenBank™ accession number GQ497342) expressed from *T. spiralis* muscle larvae. For primary characterization of the purified protein, binding affinity for fluorescent fatty acid analogue 11-[[[5-(dimethylamino)-1-naphthalenyl]sulfonyl]amino]-undecanoic acid (DAUDA) was tested by its steady-state fluorescence spectra. *Ts*-PCHTP appeared to have binding affinity of mentioned ligand but after revealing the protein sequence that affinity found to be unspecific (data not shown).

In order to identify any related proteins homology was performed by the MALDI-TOF and N-terminal sequence analyses but none was found. However, *T. spiralis* EST database search found two similar fragments and these were used to obtain the full cDNA sequence. The analysis of the amino acid sequence identified a signal peptide for extracellular localization, two homologous domains with multiple cysteine residues (pcd-1 and-2) and C-terminal histidine rich tail. Within the pcd domains a consensus motif containing cysteine and glycine/histidine residues was found (Figure 2C).

*Ts*-PCHTP pcd-1 and pcd-2 show similarity with two hypothetical *Caenorhabditis* poly-cysteine proteins, 31 kDa protein T19C3.2 (YSV2_CAEEL) and 27 kDa protein R01H10.4. These proteins form two separate groups of homology with other nematode partial protein sequences available from EST search (Figure 3), (www.nematode.net), [24]. Within these groups the homology is present predominantly at the positions of the cysteine residues. The main identity is based on the motifs ***C***x***CCC***[HPY]x[PN]x[PN]x5**C** and **CG**x**CS,** that could play a role in the metal binding. Interestingly, the *Caenorhabditis* protein R01H10.4 has an unusual poly-glutamate (**EE**RR**EEEEE**)-tail at the C-terminal end that might be a functional analogue of the metal binding histidine-tail of the *Ts*-PCHTP. Both of the *Caenorhabditis* proteins are predicted to have a signal peptide as well as *Ts*-PCHTP.

Some highly homologous EST sequences were identified from *Trichuris* species and *Trichinella pseudospiralis* (Table S1, Figure 3, Figure S3). Here the similarity is also within the conserved pcd domains. The consensus motif defined for pcd-1 and pcd-2 is found in all species mentioned above. This suggests an important role of pcd-1 and pcd-2 in the function of this protein group, most probably closely related to the metal binding properties. A histidine tail is observed at the C-terminal of all mentioned proteins. However, the signal peptides located in exon 1 are quite different between the genus *Trichuris* and *Trichinella*. We propose that the *Trichinella* and *Trichuris* proteins form a novel not yet investigated PCHTP protein family, specific for the Superfamily Trichinelloidea. Its members, successfully identified so far, are all putative proteins and are predicted to be secreted or extracellularly localized. That family is phylogenetically related to the two groups of nematode proteins similar to T19C3.2 (YSV2_CAEEL) and R01H10.4 from *Caenorabditis*.

*Ts*-PCHTP sequence analysis showed a high content of typical metal binding residues suggesting the possibility that multiple potential metal binding sites may be formed [45], [46]. Poly-histidine regions are often associated with metal ions. They might bind different divalent cations like Ni^2+^, Zn^2+^ Cu^2+^, Co^2+^, Mn^2+^, Fe^2+^, Fe^3+^. These metal binding sites might consist of either consecutive histidine residues or histidine residues in combination with one to three other amino acids in between [47], [48], [49]. In many poly-histidine regions containing proteins (for example hrg-proteins [47]) this motif is situated in the middle of the polypeptide chain and is often associated with a metal binding function. This domain could be also responsible for the Ni^2+^ found during the analysis.

Further TXRF analysis supplied evidence that *Ts*-PCHTP possesses metal binding properties. On the other hand the presence of the histidine tag as metal binding site is not sufficient to explain alone the TXRF results. In fact, almost two equivalents of nickel, iron and zinc were found per protein monomer. This suggests that other *Ts-*PCHTP sites are available for metal binding. The presence of clustered cysteines in proteins is also indicative of metal-binding domains, where metals, usually zinc, play structural role [50]. The functions of these domains include dimer formation (protein-protein interaction) and DNA binding properties (protein-nucleic acid interactions) [51], [52]. *Ts*-PCHTP contains two cysteine-rich domains, but they do not posses significant homology with any other known metal binding protein families. However, some elements in the sequence like [Cx_2_C], [C/G], [C/H] and [CCC] motifs, could be found in other protein families such as heavy metal-associated domain superfamily, P-type ATPases, metallothioneins, C2H2 zinc finger proteins and might indicate a possible localization of a potential metal binding site/s. Most cysteine rich and metal binding proteins have predominantly (-stranded structures [52], [53], which is in agreement with the fact that Ts-PCHTP is predominantly (-stranded (Figure S4). Another feature of the protein metal-binding sites is that the metal ions are often bound to hydrophilic ligands, surrounded by hydrophobic shell or situated in deep hydrophobic cavity [53]. Metalloproteins might bind specific and unspecific hydrophobic ligands like fatty acids and heme [53], [54]. As mentioned above, Ts-PCHTP showed some unspecific binding affinity for fatty acids analogue DAUDA. This ligand could bind unspecifically to a hydrophobic pocket or to the protein surface.

It is unlikely that *Ts*-PCHTP functions as a regulatory protein since it is present is such high abundance in the nematode (up to 0.2% of the total soluble proteins). The variety of bound metals found in the native protein could also explain that more probably it is involved in heavy metal storage and/or transport within the parasite. The presumption that it acts inside the parasite was confirmed by the fact that *Ts*-PCHTP possesses a signal peptide for extracellular localization, but it is not excreted into the host organism (Figure 6). It most probably functions outside the parasitic cells, in pseudocoelomic fluid or cuticle.

The investigations of *Ts*-PCHTP were performed only with the encapsulated larvae. Although the protein was not studied in adult worms the EST data for *Trichuris* species, *T. spiralis* and *T. pseudospiralis* show that similar proteins are expressed in both adult and different larvae stages (Table S1).

*Ts*-PCHTP is specific for *Trichinella* species and hence could be further studied as a potential target for diagnostics and chemotherapy.

## Supporting Information

Table S1 EST sequences related and homologous to ts-pchtp gene (www.nematode.net).
(0.06 MB PDF)

Table S2 Peptides obtained after tryptic digestion.
(0.06 MB PDF)

Figure S1 Nucleotide, derived amino acid sequence and secondary structure prediction of Ts-PCHTP. The signal peptide is underlined; two poly cysteine domains are shown in gray; histidine tail is shown in bold, italic and underlined. Secondary structure prediction by Jpred: c-coil; h-helix; e-extended (beta strand). GenBankTM accession number is GQ497342.
(0.01 MB PDF)

Figure S2 ssDNA secondary structure predicted with the mfold software (http://www.bioinfo.rpi.edu/applications/mfold). A) Folding prediction of the sense strand; B) folding prediction of the antisense strand; C) fold prediction at 37°C - self-cuted fragment shown in green and with arrows.
(4.24 MB TIF)

Figure S3 Alignment between Ts-PCHTP and composed in silico hypothetical protein sequences from EST fragments (Table S1). Identical amino acid residues are shown in gray.
(0.02 MB PDF)

Figure S4 Circular dichroism (CD) spectrum of Ts-PCHTP.
(0.39 MB PDF)
