# Supplementary material for: A Novel Secretory Poly-Cysteine and Histidine-Tailed Metalloprotein (Ts-PCHTP) from Trichinella spiralis (Nematoda)
Source: PLoS One. 2010 Oct 13;5(10):e13343. doi: 10.1371/journal.pone.0013343 (PMC2954182; doi:10.1371/journal.pone.0013343)
Supplement: Table S1 — EST sequences related and homologous to ts-pchtp gene (www.nematode.net). (0.06 MB PDF) [file pone.0013343.s001.pdf]

	Trichinella spiralis	*Trichinella pseudospiralis*	*Trichuris muris*	*Trichuris vulpis*
First domain	Contig TSC00934<br>ps20c06.y4<br>ps32b04.y1<br>ps30b10.y1<br>ps51e04.y1	FG353964.1	Contig TMC00067<br>Tm_ad_34E11_SKPL<br>Tm_ad_31C06_SKPL<br>Tm_ad_44G11_SKPL<br>Tm_ad_29G11_SKPL<br>Tm_ad_30C12_SKPL<br>MBTmAdA100SAC	ks08c12.y1
Second domain	Contig TSC01547<br>ps55f09.y1<br>MBTsMLB336SAC	FG350027.1<br>FG587443.1<br>FG355562.1<br>FG580512.1<br>FG349929.1<br>FG588442.1	Contig TMC00695<br>Tm_ad_62C05_SKPL<br>Tm_ad_10A12_SKPL<br>MBTmAdA177SAC<br>Tm_ad_07E10_SKPL<br>Tm_ad_30A06_SKPL<br>Tm_ad_31H09_SKPL<br>Tm_ad_30C07_SKPL<br>Tm_ad_28E10_SKPL	ks18e08.y1
C-end	ps95h05.y1 adult<br>ps95h01.y1 adult		Contig TMC00832<br>Tm_ad_10H11_SKPL<br>Tm_ad_57B09_SKPL<br>Tm_ad_62G07_SKPL<br>Tm_ad_33F12_SKPL<br>Tm_ad_37G10_SKPL<br>Tm_ad_31A12_SKPL<br>Tm_ad_39H07_SKPL	
