# Supplementary material for: A Novel Secretory Poly-Cysteine and Histidine-Tailed Metalloprotein (Ts-PCHTP) from Trichinella spiralis (Nematoda)
Source: PLoS One. 2010 Oct 13;5(10):e13343. doi: 10.1371/journal.pone.0013343 (PMC2954182; doi:10.1371/journal.pone.0013343)
Supplement: Table S2 — Peptides obtained after tryptic digestion. (0.06 MB PDF) [file pone.0013343.s002.pdf]

User mass	*Theoretical peptide mass*	*Δmass (Dalton)*	*Peptide*	*Position*	*Modification*
817.711	817.518	-0.192	(I)VVLFVAAV(G)	7--14	
2696.222	2696.281	0.059	(F)VAAVGFGNKISSADTCPEFG EWKPW(T)	11--35	
843.162	843.371	0.208	(K)/KEGCYPF(D)	93-99	
2415.446	2415.231	-0.214	(K)/QCLSRPDIPEYMRAGYKKLF(H)	134-153	
1377.510	1377.571	0.060	(K)/GHCIEKDNQCKC(C)	159-170	
1624.812	1624.544	0.268	DNQCK	165-169	glycosylated
1249.329	1249.462	-0.132	DNQCK	165-169	glycosylated
1377.078	1377.261	-0.182	DNQCK	165-169	glycosylated
1222.285	1222.483	0.198	(Q)CAPFNEPGDWS(E)	192-202	
568.456	568.259	-0.197	(E)CLWF(P)	204-207	
568.638	568.259	-0.379	(E)CLWF(P)	204-207	
1248.962	1248.673	-0.289	(R)/LKCQSREKKE(G)	252-261	
920.747	920.523	-0.223	(P)LKVDKKSC(G)	266-273	
1041.203	1041.332	0.129	(A)EDCPTCGDVC(T)	276-285	
1896.619	1896.674	-0.054	QNNSCAFTK	290-298	glycosylated
1700.337	1377.261	-0.215	QNNSCAFTK	290-298	glycosylated
1566.879	1566.686	-0.192	(L)PYGHCKEVGDKCKC(C)	333-346	
871.144	871.387	0.242	(Q)YCKSLEE(V)	365-371	
