# Supplementary material for: A Novel Secretory Poly-Cysteine and Histidine-Tailed Metalloprotein (Ts-PCHTP) from Trichinella spiralis (Nematoda)
Source: PLoS One. 2010 Oct 13;5(10):e13343. doi: 10.1371/journal.pone.0013343 (PMC2954182; doi:10.1371/journal.pone.0013343)
Supplement: Figure S1 — Nucleotide, derived amino acid sequence and secondary structure prediction of Ts-PCHTP. The signal peptide is underlined; two poly cysteine domains are shown in gray; histidine tail is shown in bold, italic and underlined. Secondary structure prediction by Jpred: c-coil; h-helix; e-extended (beta strand). GenBankTM accession number is GQ497342. (0.01 MB PDF) [file pone.0013343.s003.pdf]

5` end

```
1       AAAAAAGTGCCCCTCAACAGCAGCACTGCAAAAAGAAAAGCAGATatggctttctcaact
                                                  M A F S T
                                                  c c h h h
61      attgtcgtgctttttgttgcagccgttggtttcggtaacaaaatttcgtcggccgacaca
         I V V L F V A A V G F G N K I S S A D T
         h e e e e e e h h c c c c c c c c c c c
121     tgtcccgaatttggagaatggaaaccttggacagaatgtttatggtatccgatgcagaat
         C P E F G E W K P W T E C L W Y P M Q N
         c c c c c c c c c c c h h c c c c h h h
181     atttacgacaaaatgaccgccagttgtggtttgcctggacatcgcaatttgacgaacatt
        I Y D K M T A S C G L P G H R N L T N I
        h h h h h h h c c c c c c c c c c h c c
241     ttgcctctaccaccgggctttactatccctccaccttgtggtcattgcagtttcaagacc
         L P L P P G F T I P P P C G H C S F K T
         c c c c c c c c c c c c c c c c c c h h
301     cgctgtcgtacacgtccgaagaaggaaggatgctacccattcgacggtgaacgagaaatc
         R C R T R P K K E G C Y P F D G E R E I
         h c c c c c c c c c c c c c c c c c h h
361     tgccacgaacatggcgatatttgcaccatagctaaactgccaggaattggctgcggttgg
         C H E H G D I C T I A K L P G I G C G W
         h c c c c c e e e e h c c c c c c c c h
421     acagttttgcaagaagtggtcaagcagtgtttgtccagacctgacatacctgaatatatg
         T V L Q E V V K Q C L S R P D I P E Y M
         h h h h h h h h h h h c c c c c h h h h
481     agagctggttataagaagctgttccacatgttgcccaaaggccattgtattgagaaagat
         R A G Y K K L F H M L P K G H C I E K D
         h h h h h h h h h c c c c c c c c c c c
541     aatcagtgcaaatgttgctgcggagattacgaacccaacgaagatggaactgaatgtgtt
         N Q C K C C C G D Y E P N E D G T E C V
         c c c c c c c c c c c c c c c c c c h h
601     aaacaacaagatcatcagtgtgccccgttcaatgaacccggagattggagtgaatgtttg
         K Q Q D H Q C A P F N E P G D W S E C L
         h c c c c c c c c c c c c c c h h h h c
661     tggttcccgttggcggatatgttcaagaaggtgcaaagccattgcggtgtcgaaggcaag
         W F P L A D M F K K V Q S H C G V E G K
         c c c h h h h h h h c c c c c c c c c c
721     cctgaaggtctgtctccaagttcgctggcaccggccggatttcagattccggagaagtgc
         P E G L S P S S L A P A G F Q I P E K C
         c c c c c c c c c c c c c c c c c c c c
781     ggcttttgctcgttccgcttgaaatgccaaagtcgagagaagaaagagggatgcttcccg
         G F C S F R L K C Q S R E K K E G C F P
         c c c c h h h c c c c c c c c c c c c c
841     ttgaaggtggataagaaaagttgcggcgctgaagactgtccaacttgcggagatgtgtgc
         L K V D K K S C G A E D C P T C G D V C
         c c c c c c c c c c c c c c c c c c c c
901     accttggacaagcagaacaacagctgcgcgtttacaaaggcaatgggaatgaaattctgg
         T L D K Q N N S C A F T K A M G M K F W
         c c c c c c c c c h h h h h c c c c c c
961     aacagctttgcgcacaaagcaaaagaaagcaatttagctcactggcgtcgtgatggttat
         N S F A H K A K E S N L A H W R R D G Y
         c c c h h h h c c c c h h h h h h h h h
1021    gcggatctgttcaaatttttaccatacggtcattgcaaagaagtgggcgacaaatgcaaa
         A D L F K F L P Y G H C K E V G D K C K
         h h h h h c c c c c c c c c c c c h h h
1081    tgctgctgtcatccgtatgaaccgaacgaagacggcactgcttgtgtagtgaagcaatat
         C C C H P Y E P N E D G T A C V V K Q Y
         h c c c c c c c c c c c h h h h h h h h
1141    tgtaaatcgttggaagaggtaggaggcaaaaagcagcagaaagatcagccagaaagtgaa
         C K S L E E V G G K K Q Q K D Q P E S E
         h h h h h h c c c c c c c c c c c c h h
1201    aagaaagctgaaaatatgcccgaaactactggaaacgcatcccatcatcagcatcgccat
         K K A E N M P E T T G N A S H H Q H R H
         h h h c c c c c c c c c c c c c c c c c
1261    catcacggcgacagtagcagcgaaagtcacgaacaacatcatcatcatcatcatcattga
         H H G D S S S E S H E Q H H H H H H -
         c c c c c c c c c h h h c c c c c c
1321    AAGCAGAAAAATATTTCGTCTCCTTCCTGACTTAGAGTTTTTGAATATTATTGCTTTGCT
1381    TTAGTCATAAAATCAACGCTCATTAATTATTATATCATGCAGTTTCATGGTTGGAGGCAT
1441    AACATATAAATATATACAATTATTATATTTTCATATATGATTTTCAGTGACACATTGAAA
1501    TTATTCAGAATTGATCAACATTTAAAAACATTCGTTCATCACTGTAGTTTGGGGCTTATT
1561    AAAAATGCATTCATTT
```

3`end
