# Supplementary figures and images for: A Novel Secretory Poly-Cysteine and Histidine-Tailed Metalloprotein (Ts-PCHTP) from Trichinella spiralis (Nematoda)
Source: PLoS One. 2010 Oct 13;5(10):e13343. doi: 10.1371/journal.pone.0013343 (PMC2954182; doi:10.1371/journal.pone.0013343)

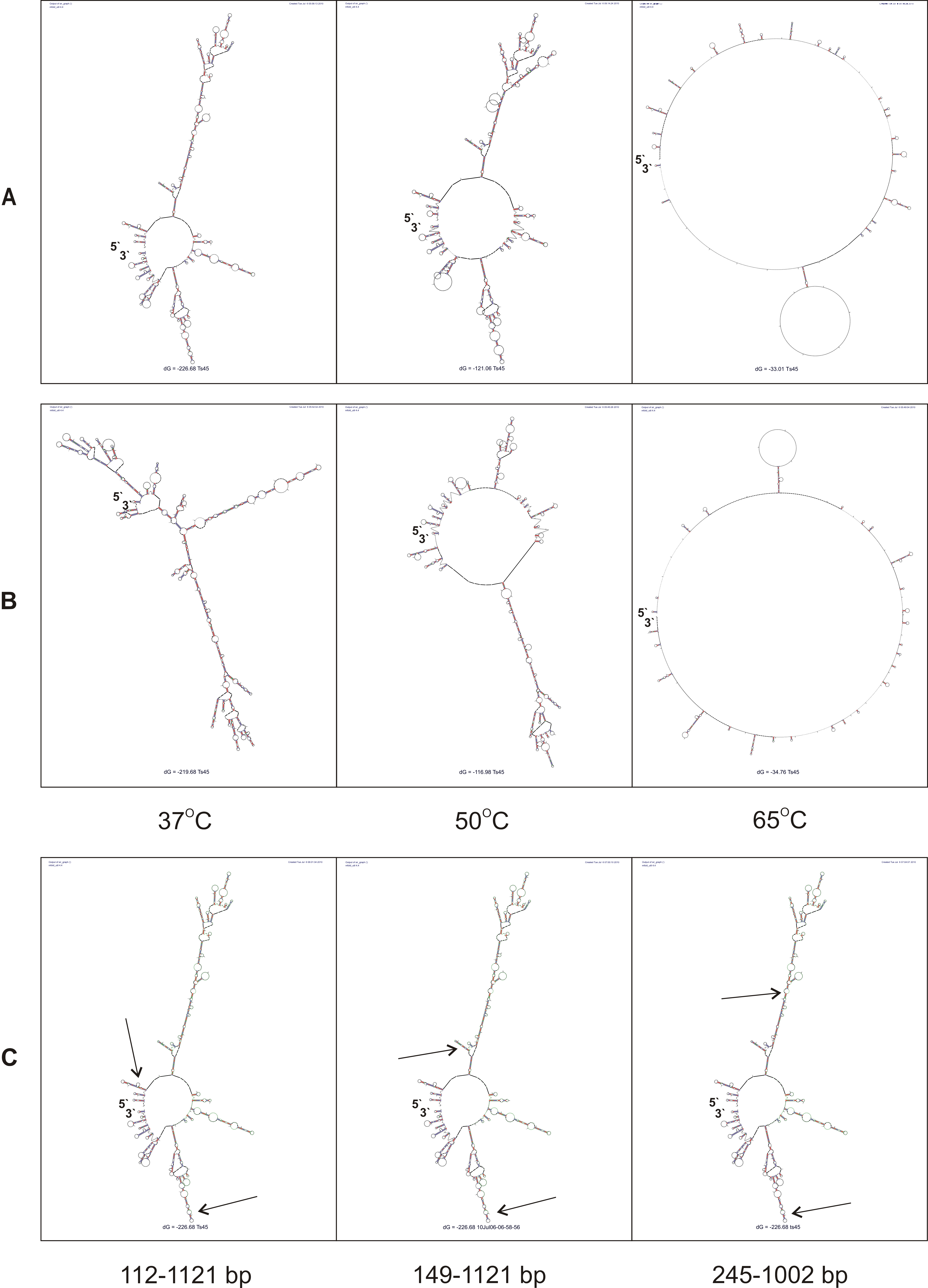

Supplement: Figure S2 — ssDNA secondary structure predicted with the mfold software (http://www.bioinfo.rpi.edu/applications/mfold). A) Folding prediction of the sense strand; B) folding prediction of the antisense strand; C) fold prediction at 37°C - self-cuted fragment shown in green and with arrows. (4.24 MB TIF) [file pone.0013343.s004.tif]
