# Supplementary material for: A Novel Secretory Poly-Cysteine and Histidine-Tailed Metalloprotein (Ts-PCHTP) from Trichinella spiralis (Nematoda)
Source: PLoS One. 2010 Oct 13;5(10):e13343. doi: 10.1371/journal.pone.0013343 (PMC2954182; doi:10.1371/journal.pone.0013343)
Supplement: Figure S3 — Alignment between Ts-PCHTP and composed in silico hypothetical protein sequences from EST fragments (Table S1). Identical amino acid residues are shown in gray. (0.02 MB PDF) [file pone.0013343.s005.pdf]

```
T.spiralis          MAFSTIVVLFVAAVGFGNKISSADTCPEFGEWKPWTECLWYPMQNIYDKMTASCGLPGHR
T.pseudospiralis    --------------------------PEFGEWKPWTECLWYPMQNIYDKMTASCGLHGHR
Trichuris muris     ---AMLALFFPLLLTVRLSTAGHVKCPDFGDWKPWTDCLWYPPQHMYSKLSHACGMHAHR
Trichuris vulpis    -MAAMLLLFLPLLLAVGPSTAGHVKCPDFGDWKPWTDCLWYPPQNMYSKLAHACGMHAHR
                    -------------------------------------C---------------C------

T.spiralis          NLTNILPLPPGFTIPPPCGHCSFKTRCRTRPKKEGCYPFDGEREICHEHGDICTIAKLPG
T.pseudospiralis    NLTNILPLPPGFTIPPPCGHCSFKTRCRTRPKKEGCYPFDGEREICHEHGDICTISKLPG
Trichuris muris     NLTGVMDLPHGHKTPPPCGHCSFKFRCRRRPNTEGCYPLDGEVEVCHDHSDICTLPKLPH
Trichuris vulpis    NLTGVMDLPHGHKTPPPCGHCSFKFRCRRRPNTEGCYPLDGEVEVCHDHSDICTLPKLPH
                    -----------------C--C-----C--------C---------C------C-------

T.spiralis          IGCGWTVLQEVVKQCLSRPDIPEYMRAGYKKLFHMLPKGHCIEKDNQCKCCCGDYEPNED
T.pseudospiralis    IGCGWTILHEVVG-----------------------------------------------
Trichuris muris     LGCGYAFINEKLKQCFTRPDTPSYVRLGYRKMFESIPKKHCIEKDGMCKCCCGDYEPNES
Trichuris vulpis    LGCGYAFINEKLKQCFARPDMPSYVRLGYRKMFESIPKKHYIEKDGMCKCCCGDYEPNES
                    --C-----------C-------------------------C------C-CCC--------

T.spiralis          GTECVKQQDHQCAPFNEPGDWSECLWFPLADMFKKVQSHCGVEGKPEGLSPSSLAPAGFQ
T pseudospiralis    ------------------------------------------------------------
Trichuris muris     GTECIKPPAHDCPAYGPPSEWSECLWFPLKNIVSHVYDHCHVHKEPDGYEPHSVAPANVH
Trichuris vulpis    GTECIKPPAHDCPAYGPPSEWSECLWFPLKNIVSHVYDHCHVHKEPDGYEPHSVAPANLH
                    ---C-------C-----------C---------------C--------------------

T.spiralis          IPEKCGFCSFRLKCQSREKKEGCFPLKVDKKSCGAEDCPTCGDVCTLDKQNNSCAFTKAM
T.pseudospiralis    ---------------------------------GAEDCPTCGDVCTLDKQNNSCAFTKAM
Trichuris muris     IPEKCGFCSFRVKCMKRDKKDGCFPLKLGKKSCGKDDCPTCGDICTLDKINGSCAFPRVM
Trichuris ulpis     VPEKCGYCSFRVKCMKRDKKDGCFPLKLGKKNCGKDDCPTCGDICTLDKINGSCVYPRVM
                    -------C-----C--------C---------C----C--C---C--------C------

T.spiralis          GMKFWNSFAHKAKESNLAHWRRDGYADLFKFLPYGHCKEVGDKCKCCCHPYEPNEDGTAC
T.pseudospiralis    GMKFWNSFAHKAKESNLAHWRRDGYADLFKFLPYGHCKEVGDKCKCCCHPYEPNEDGTAC
Trichuris muris     KEKIWDDFTATSKEKHMPHWKRDGYAKMLMQLPYSNCKEVGDKCKCCCHPYEPNKDGTAC
Trichuris vulpis    KEKIWDDFTATSKEKHMPHWKRDGYAKMLMQL----------------------------
                    -------------------------------------------C-CCC-----------C

T.spiralis          VVKQYCKSLEEVGGKKQQKDQPESEKKAENMPETTGNASHHQHRHHHGDSSSESHEQHHHHHHH
T.pseudospiralis    ILKQYCKSLEEIGDKKQLQDQPDGEKKAENMPETSGNSSHHHHRHHHGDSSSESHERHHQHH--
Trichuris muris     VVKEYCKRVHEL----------------------HHHDHHGHGEEHHKSSSSESKEHHHH----
Trichuris vulpis    ----------------------------------------------------------------
                    -----C----------------------------------------------------------
```
