# Supplementary material for: A Novel Secretory Poly-Cysteine and Histidine-Tailed Metalloprotein (Ts-PCHTP) from Trichinella spiralis (Nematoda)
Source: PLoS One. 2010 Oct 13;5(10):e13343. doi: 10.1371/journal.pone.0013343 (PMC2954182; doi:10.1371/journal.pone.0013343)
Supplement: Figure S4 — Circular dichroism (CD) spectrum of Ts-PCHTP. (0.39 MB PDF) [file pone.0013343.s006.pdf]

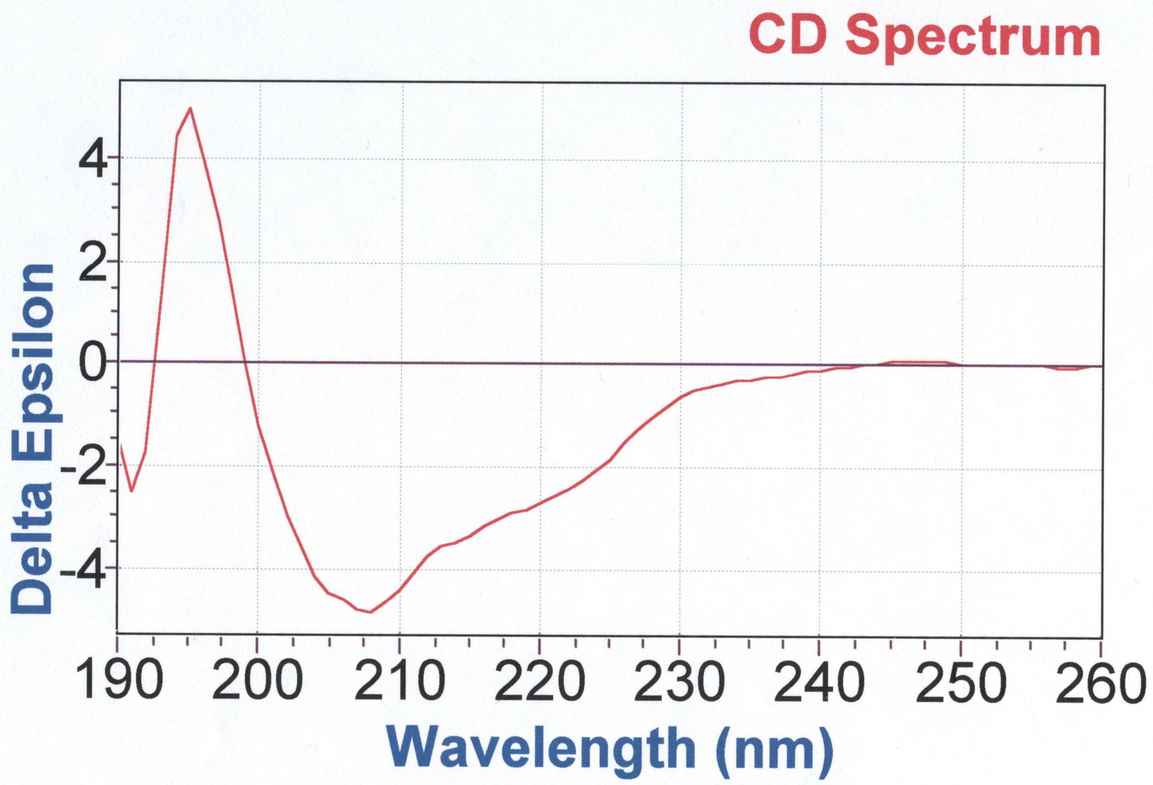

	180-260nm	185-260nm	190-260nm	195-260nm	200-260nm	205-260nm	210-260nm
Helix	n.d.	n.d.	21.1 %	25.7 %	21.4 %	21.0 %	24.8 %
Antiparallel	n.d.	n.d.	11.3 %	15.6 %	13.9 %	14.0 %	16.0 %
Parallel	n.d.	n.d.	4.0 %	6.0 %	4.9 %	5.3 %	5.7 %
Beta-Turn	n.d.	n.d.	29.1 %	22.0 %	23.1 %	25.8 %	18.8 %
Rndm. Coil	n.d.	n.d.	35.8 %	29.3 %	34.1 %	33.5 %	33.8 %
Total Sum	-	-	101.2 %	98.6 %	97.3 %	99.7 %	99.1 %
